# 2355. Incidence of Omicron Variant Reinfection and Reduction of Reinfection Risk After Coronavirus Disease 2019 Vaccination in Children

**DOI:** 10.1093/ofid/ofad500.1976

**Published:** 2023-11-27

**Authors:** Tatsuki Ikuse, Yuta Aizawa, Satoshi Hasegawa, Masashi Takahashi, Takanori Hayashi, Miyako Kon, Tsutomu Tamura, Haruki Matsumoto, Akihiko Saitoh

**Affiliations:** Niigata University Graduate School of Medical and Dental Sciences, Niigata, Niigata, Japan; Niigata University Graduate School of Medical and Dental Sciences, Niigata, Niigata, Japan; Niigata Prefectural Office, Niigata, Niigata, Japan; Niigata Prefectural Office, Niigata, Niigata, Japan; Niigata Prefectural Institute of Public Health and Environmental Science, Niigata, Niigata, Japan; Niigata Prefectural Institute of Public Health and Environmental Science, Niigata, Niigata, Japan; Niigata University Graduate School of Medical and Dental Sciences, Niigata, Niigata, Japan; Niigata Prefectural Office, Niigata, Niigata, Japan; Niigata University, Niigata, Niigata, Japan

## Abstract

**Background:**

Reinfection by severe acute respiratory syndrome coronavirus 2 (SARS-CoV-2) has become an important concern after the emergence of SARS-CoV-2 omicron variants (e.g., BA.1, BA.2, and BA.5). Reinfection by variants can occur in children; however, data are limited on the incidence of reinfection and the effectiveness of coronavirus disease 2019 (COVID-19) vaccines against reinfection.

**Methods:**

This population-based cohort study collected clinical data for COVID-19 from children aged 15 years or younger who were diagnosed as having COVID-19 in Niigata, Japan, from January through August 2022, when omicron variants dominated. We analyzed three periods with different predominant variants (BA.1, BA.2, and BA.5). Reinfection was monitored during each period and observation was extended through November 2022. We calculated the incidence of reinfection and the hazard ratio (HR) for reinfection in relation to COVID-19 vaccination status.

**Results:**

During the study period, 1338/48,099 children (2.8%) were reinfected by omicron variants; most reinfections occurred in the BA.5-predominant period (1265/1338, 94.5%). The median interval to reinfection was 169 days (interquartile range 120-207 days) (**Table 1**). Among children eligible for COVID-19 vaccination, the vaccine was effective in preventing reinfection: the HR for reinfection in children who completed vaccination was 0.29 (95% confidence interval, 0.20–0.40) (**Figure 1**).

**Table 1.**
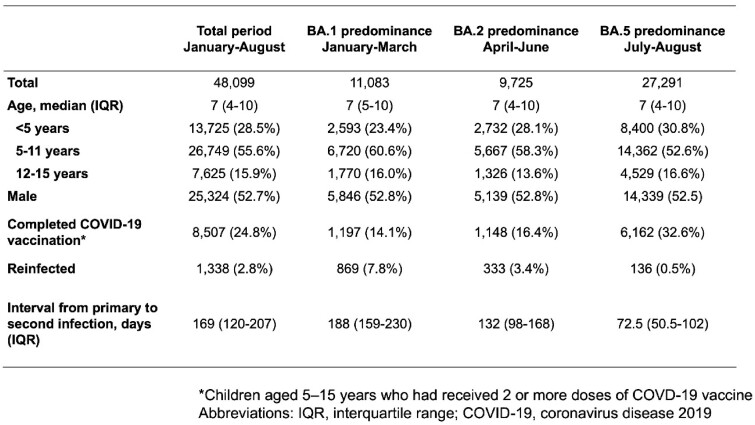
Characteristics of children with primary coronavirus disease 2019 infection during periods of BA.1-, BA.2-, and BA.5-predominance in 2022 in Niigata, Japan

**Figure 1.**
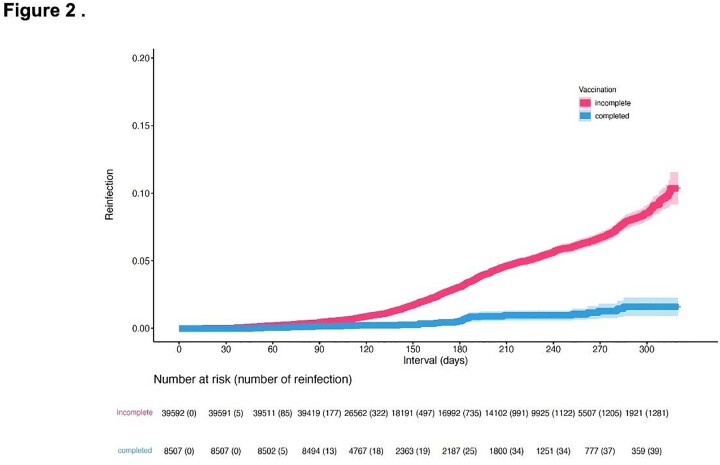
Prevalence of pediatric COVID-19 reinfection after complete and incomplete vaccination

The prevalence of reinfection by omicron was analyzed by using Kaplan–Meier analysis. Children aged 5–15 years were classified on the basis of COVID-19 vaccination history before primary infection. The hazard ratio for reinfection among children aged 5–15 years with a history of complete COVID-19 vaccination before primary infection was 0.21 (95% confidential interval 0.16-0.29), as compared with those with a history of incomplete vaccination.

**Conclusion:**

Reinfection in children was frequent when omicron variant BA.5 was predominant in Japan. Children who underwent COVID-19 vaccination had a significantly lower risk of omicron reinfection, which suggests that vaccination is important in preventing reinfection by SARS-CoV-2 omicron variants and preparing for outbreaks of new variants.

**Disclosures:**

**All Authors**: No reported disclosures

